# Particulate Matter and Its Molecular Effects on Skin: Implications for Various Skin Diseases

**DOI:** 10.3390/ijms25189888

**Published:** 2024-09-13

**Authors:** Kyungho Paik, Jung-Im Na, Chang-Hun Huh, Jung-Won Shin

**Affiliations:** 1 Department of Dermatology, Seoul National University Bundang Hospital, Seongnam 13620, Republic of Korea; ndrdzdr@gmail.com (K.P.); vividna@gmail.com (J.-I.N.); chhuh@snu.ac.kr (C.-H.H.); 2 Department of Dermatology, Seoul National University College of Medicine, Seoul 03080, Republic of Korea

**Keywords:** particulate matter, skin disease, atopic dermatitis, psoriasis, skin aging

## Abstract

Particulate matter (PM) is a harmful air pollutant composed of chemicals and metals which affects human health by penetrating both the respiratory system and skin, causing oxidative stress and inflammation. This review investigates the association between PM and skin disease, focusing on the underlying molecular mechanisms and specific disease pathways involved. Studies have shown that PM exposure is positively associated with skin diseases such as atopic dermatitis, psoriasis, acne, and skin aging. PM-induced oxidative stress damages lipids, proteins, and DNA, impairing cellular functions and triggering inflammatory responses through pathways like aryl hydrocarbon receptor (AhR), NF-κB, and MAPK. This leads to increased production of inflammatory cytokines and exacerbates skin conditions. PM exposure exacerbates AD by triggering inflammation and barrier disruption. It disrupts keratinocyte differentiation and increases pro-inflammatory cytokines in psoriasis. In acne, it increases sebum production and inflammatory biomarkers. It accelerates skin aging by degrading ECM proteins and increasing MMP-1 and COX2. In conclusion, PM compromises skin health by penetrating skin barriers, inducing oxidative stress and inflammation through mechanisms like ROS generation and activation of key pathways, leading to cellular damage, apoptosis, and autophagy. This highlights the need for protective measures and targeted treatments to mitigate PM-induced skin damage.

## 1. Introduction

Air pollution caused by rapid industrialization and its effect on human health has become a major global concern. Particulate matter (PM) is a major harmful air pollutant composed of a complex mixture of solid and liquid particles that vary in size [1,2]. PM originates from both natural and anthropogenic sources. It includes several chemical constituents, such as nitrates, sulfates, elemental and organic carbon, organic compounds (e.g., polycyclic aromatic hydrocarbons [PAHs]), biological compounds (e.g., endotoxins, cell fragments), and metals (e.g., iron, copper, nickel, zinc, and vanadium) [3]. Based on size, PM is classified as ultrafine (particles with a diameter < 0.1 µm), fine (particles with a diameter < 2.5 µm: PM2.5), and coarse (particles with a diameter < 10 µm: PM10). PM can penetrate the airways from the nasal passages to the alveoli where gas exchange occurs, leading to numerous adverse health effects [3]. PM induces pro-inflammatory effects by generating reactive oxygen species (ROS), leading to oxidative stress [4,5]. ROS production triggers the release of inflammatory cytokines, resulting in inflammation and tissue damage [6,7]. PM can cause irregular heartbeat, aggravated asthma, decreased lung function, and increased respiratory symptoms, as well as premature death in individuals with heart or lung disease [8,9,10].

As the outermost layer of the body, the skin is at a greater risk of exposure to PM. Unlike the respiratory mucosa, the skin has a stratum corneum composed of dead keratinocytes embedded in a lipid matrix, serving as the strongest barrier against environmental stressors, including PM. However, a previous study demonstrated that PM could penetrate barrier-disrupted skin and hair follicles [11]. Moreover, PM is associated with the aggravation of skin disorders, including atopic dermatitis (AD), eczema, skin aging, and pigmentation [12,13,14]. With increasing evidence, numerous review articles have emerged that summarize the effects of PM on various skin conditions [2,12,15,16]. However, most existing reviews have primarily focused on epidemiologic findings. Although some studies have explored biological mechanisms, none have systematically organized how PM contributes to the specific pathogenesis of individual diseases. This review explores the relationship between PM and various skin diseases and examines the molecular mechanisms involved, including disease-specific pathways.

## 2. Methodology

We performed a comprehensive literature search across databases including PubMed, Medline, and Scopus. Initially, we used key search terms such as “particulate matter” combined with “skin diseases” or “skin health” to identify specific skin conditions associated with PM exposure. Epidemiological studies, narrative reviews, and systematic reviews were included in this phase. Based on the strength of the evidence, four skin conditions—atopic dermatitis, psoriasis, acne, and skin aging—were selected. Subsequently, we conducted a second search using “particulate matter” in combination with terms like “skin disease”, “skin physiology”, “atopic dermatitis”, “psoriasis”, “acne”, “skin aging”, and “skin pigmentation.” At this stage, only laboratory studies using skin cells or review articles summarizing laboratory findings were included.

## 3. Basic Molecular Mechanisms of Particulate Matter (PM)-Induced Skin Damage

PM primarily induces skin diseases through oxidative stress, which leads to inflammatory responses and barrier disruption [15]. A significant concern is whether PM can penetrate the skin barrier and reach viable cell layers, thereby affecting skin biology. PM can penetrate the epidermis through hair follicles or a disrupted stratum corneum [11]. Although PM particles do not penetrate, they adhere to the skin surface and release soluble components, such as gases or ions, causing harmful effects [11,15]. This section describes the general molecular mechanisms underlying PM-induced skin damage.

### 3.1. Penetration of PM into the Skin

**Figure 1:**
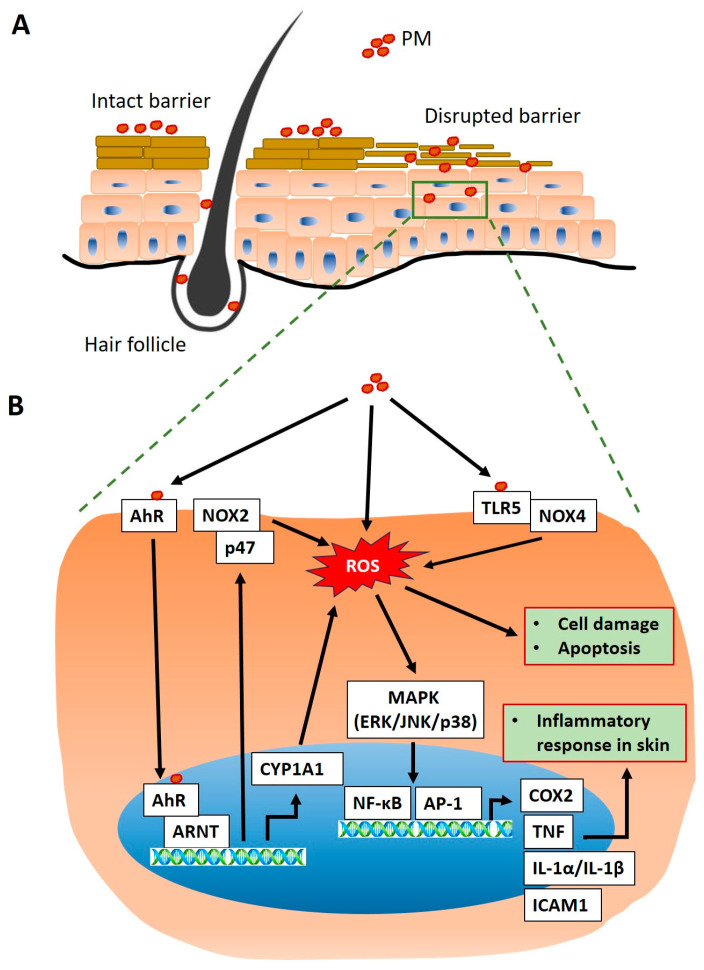
Primary molecular mechanisms of PM-induced skin damage. (**A**) PM can penetrate the skin, infiltrating both the barrier-disrupted interfollicular epidermis and the intact follicular epidermis. (**B**) PM activates cellular signaling pathways such as AhR and TLR, leading to increased ROS production, while PM itself also generates ROS. ROS from PM causes oxidative stress, damaging lipids, proteins, and DNA, impairing cellular functions, and causing apoptosis. It activates NF-κB, promoting cytokines (TNF, IL-1α, IL-1β), adhesion molecules (ICAM1), and enzymes (COX2). ROS also activate MAPK pathways (ERK, JNK, p38), leading to inflammatory responses in skin.

A study using a mouse model found that PM could penetrate the skin following barrier disruption induced by tape stripping, infiltrating both the barrier-disrupted interfollicular epidermis and the intact follicular epidermis, resulting in cutaneous inflammation (Figure 1A) [11]. In addition, multimodal nonlinear optical imaging in an ex vivo human skin model showed deeper PM penetration with increased tape stripping, leading to higher pro-inflammatory cytokine secretion [17]. Another study using a reconstructed human epidermis model demonstrated that PM particles penetrated the stratum corneum after 24 h and reached deeper layers after 48 h [18].

### 3.2. Aryl Hydrocarbon Receptor Activation

The aryl hydrocarbon receptor (AhR) is a ligand-activated transcription factor that mediates the skin response to environmental pollutants, including PM IL (Figure 1B) [19]. AhR is ubiquitously expressed in all skin cell types and plays crucial roles in epidermal differentiation, barrier function, and the maintenance of skin homeostasis [20,21,22]. PAHs, the components bound to the surface of PM, act as ligands for AhR. Upon binding, AhR is activated and translocated to the nucleus, where it binds to the AhR nuclear translocator (ARNT) [23]. The AhR–ligand–ARNT complex promotes the transcription of the cytochrome p450 (CYP) family, including CYP1A1, CYP1A2, and CYP1B1 [24]. CYP enzymes metabolize PAHs, and the resulting metabolites can cause cell damage through the formation of DNA and protein adducts or ROS generation. PM exposure upregulates CYP mRNA and protein expression, both in vitro and in vivo [18,23,25,26,27,28,29]. Additionally, PM can induce ROS production via the AhR/p47 phox/nicotinamide adenine dinucleotide phosphate hydrogen (NADPH) oxidase (NOX)2 pathway [30,31,32,33,34].

### 3.3. Oxidative Stress Induced by PM

Oxidative stress is one of the primary mechanisms through which PM harms the skin. ROS, which are highly reactive molecules involved in oxygen metabolism, play a central role in this process (Figure 1B) [35]. PM induces ROS generation directly and indirectly. When antioxidant defenses of the skin, such as superoxide dismutase, catalase, and glutathione peroxidase, are overwhelmed by excessive ROS, oxidative stress is induced [36].

#### 3.3.1. Reactive Oxygen Species Formation by PM

PM directly induces the production of ROS through redox-active components, such as transition metals, which catalyze ROS generation [37]. PM surfaces contain organic compounds, such as PAHs, that form quinones upon metabolic activation by CYP enzymes [38]. These quinones undergo redox cycling, leading to the production of superoxide anion radicals and hydrogen peroxide in the skin cells. PM also indirectly induces ROS production by activating cellular signaling pathways. A key pathway is the generation of ROS through the AhR, as previously mentioned. Additionally, pattern recognition receptors, such as toll-like receptors (TLRs) on keratinocytes, recognize PM and initiate intracellular signaling. PM induces a direct interaction between TLR5 and NOX4, leading to the production of ROS and the activation of the nuclear factor kappa-light-chain-enhancer of activated B cells (NF-κB)–interleukin (IL)-6 pathway. This signaling cascade results in the expression of inflammatory cytokines, which contribute to the inflammatory response and exacerbate skin damage [31]. Additionally, PM exposure disrupts mitochondrial function, causing electron leakage from the electron transport chain and enhancing intracellular ROS levels [15]. Additionally, PM-induced endoplasmic reticulum (ER) stress contributes to ROS production by disrupting protein folding and inducing an unfolded protein response [39,40,41].

#### 3.3.2. Lipid Peroxidation, Protein Oxidation, and DNA Damage

ROS, particularly hydroxyl radicals, react with lipids in cell membranes, initiating a chain reaction that damages polyunsaturated fatty acids. This process generates lipid peroxide and reactive aldehydes, such as malondialdehyde and 4-hydroxynonenal. Lipid peroxidation compromises cell membrane integrity and fluidity and impairs cellular functions and signaling pathways. Oxidative damage affects the barrier function of the skin, leading to increased permeability and susceptibility to environmental aggressors and allergens [42,43].

Additionally, ROS, including hydroxyl radicals and superoxide anions, oxidize amino acid residues in proteins, resulting in the formation of carbonyl groups and disulfide bonds. This oxidative modification affects the protein structure and function, leading to the formation of protein aggregates and degradation products. Protein oxidation impairs critical cellular functions and disrupts enzymatic activity, receptor signaling, and structural integrity. In the skin, oxidative damage can compromise the extracellular matrix (ECM) and cellular cytoskeleton, leading to skin conditions [44].

PM-induced ROS production can also lead to significant DNA damage in the skin cells. ROS can directly attack DNA molecules, causing strand breaks, base modifications, and cross-linking [45]. Oxidative DNA damage activates cellular repair mechanisms and stress responses; however, excessive or persistent damage can overwhelm these systems, resulting in mutations and apoptosis that can cause skin diseases [15,39].

#### 3.3.3. PM-Induced Cell Death by Mitochondrial Damage and Endoplasmic Reticulum Stress

Oxidative stress induced by PM can lead to the activation of apoptosis and programmed cell death [46]. Excessive ROS disrupt the electron transport chain of the mitochondria, causing electron leakage and further ROS production. Oxidative stress results in mitochondrial membrane disruption, mitochondrial DNA damage, and impaired ATP synthesis [18,39,47]. It also leads to the release of proapoptotic factors, such as cytochrome C, and the activation of caspase 3, caspase 9, and poly (ADP-ribose) polymerase [39,41,48,49,50]. These events exacerbate injury and promote the apoptosis of skin cells, further contributing to skin damage. In addition, PM-induced ROS causes ER stress by interfering with protein folding and homeostasis [39,51]. PM2.5 upregulates the protein levels of CCAAT enhancer-binding protein homologous protein, a transcription factor that mediates ER stress-induced apoptosis [39,41,52]. ER stress is also associated with autophagy. PM exposure upregulates light-chain 3B II, a protein involved in the initiation of autophagosome formation [26,39].

### 3.4. Inflammatory Responses

Activation of the inflammatory cascade is another crucial mechanism by which PM affects skin diseases via ROS production (Figure 1B). PM-induced ROS can activate NF-κB, a key transcription factor that induces a large number of inflammatory genes [53,54]. Its targets include chemokines and cytokines, such as tumor necrosis factor (TNF), IL 1-alpha (IL-1α), IL-1 β, and C-X-C motif chemokine ligand (CXCL8), crucial for skin inflammation [25,55]. Other targets include adhesion molecules, such as intercellular adhesion molecule 1 (ICAM1), and enzymes, such as cyclooxygenase (COX)2 and inducible nitric oxide synthase (iNOS) [18,27,55,56]. COX2 is essential for prostaglandin E2 formation from arachidonic acid in membrane phospholipids [57]. In addition, PM-induced ROS can activate mitogen-activated protein kinase (MAPK) and its members, including extracellular signal-regulated kinase (ERK), c-Jun N-terminal kinase (JNK), p38, and transcription factor adaptor protein 1 [58]. The exposure of fibroblasts and keratinocytes to PM induces the MAPK pathway through the phosphorylation of ERK, JNK, and p38 [32,33,53,59]. PM exposure also stimulates inflammasome activation. A study has shown that PM-treated human immortalized epidermal cells exhibit elevated mRNA levels of NOD-like-receptor containing a pyrin domain 1 (NLRP1), an inflammasome-related gene, and increased protein levels of IL-1β, a key downstream factor of NLRP1 [60]. This activation contributes to an inflammatory response in the skin, further exacerbating skin diseases and damage.

### 3.5. Summary

PM can penetrate disrupted and intact skin, causing inflammation It induces ROS generation both directly and indirectly. Directly, PM itself produces ROS. Indirectly, PM activates cellular signaling pathways such as AhR and TLR, leading to increased ROS production. ROS from PM overwhelm skin defenses, causing oxidative stress, damaging lipids, proteins, and DNA, and impairing cellular functions. PM exposure activates NF-κB complexes, promoting cytokines (TNF, IL-1α, IL-1 β, CXCL8), adhesion molecules (ICAM1), and enzymes (COX2, iNOS). ROS also activate MAPK pathways (ERK, JNK, p38), increasing inflammation and leading to mitochondrial and ER stress, resulting in apoptosis and autophagy.

## 4. Skin Diseases Associated with PM and Disease-Specific Molecular Mechanisms

**Figure 2:**
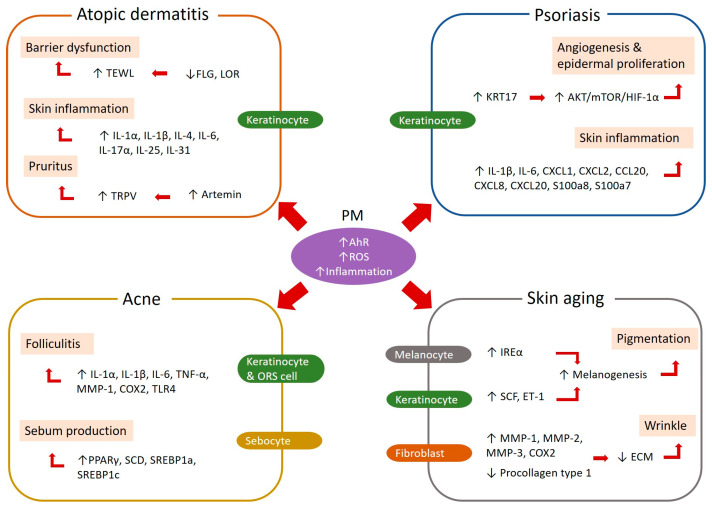
A schematic representation of the molecular effects of particulate matter on skin diseases, illustrating the disease-specific pathways involved. TEWL = transepidermal water loss, FLG = filaggrin, LOR = loricrin, IL = interleukin, TRPV = transient receptor potential vanilloid, AKT = protein kinase B, mTOR = mammalian target of rapamycin, HIF-1a = hypoxia inducible factor 1 subunit alpha, CCL, CXCL = chemokine ligand, IRE1α = Inositol-requiring transmembrane, kinase/endoribonuclease 1α, SCF = stem cell factor, ET-1 = endothelin 1, MMP = matrix metalloproteins, COX = cyclooxygenase, ECM = extracellular matrix.

Numerous epidemiological studies have demonstrated that PM is associated with various skin conditions (Table 1). Additionally, several experimental studies have proposed potential mechanisms by which PM affects the pathogenesis of specific skin diseases (Table 2, Figure 2). This section explores how PM influences disease-specific pathways at the molecular level for each condition, focusing on representative skin diseases that have been epidemiologically related to PM exposure.

### 4.1. Atopic Dermatitis

AD, also known as atopic eczema, is a chronic relapsing inflammatory skin disorder characterized by intense pruritus, erythema, scaling, and lichenification. It predominantly affects the flexural areas in children and adults and the face and extensor surfaces of infants. Both genetic predisposition and environmental factors are known to be contributing factors in the development of AD [100,101]. To date, most studies on PM and skin diseases have focused on AD (Table 1). In a prospective cohort study using the UK Biobank, PM2.5 absorbance (black carbon) had a substantial effect on the incidence of adult-onset AD [61]. Other studies have also shown that increased levels of PM10 and/or PM2.5 are positively associated with increased patient visits for AD [65,66,102]. However, some studies have shown no association between PM and AD. For example, PM2.5 was not associated with AD when adjusting for nitrogen dioxide in a study conducted in the USA [62].

The key pathogenesis of AD centers on skin barrier dysfunction and immune dysregulation. Skin barrier dysfunction is primarily associated with genetic abnormalities, particularly in the essential membrane protein filaggrin (FLG). Immune dysregulation in AD is characterized by a dominant Th2 axis involving cytokines such as IL-4, IL-13, IL-5, thymic stromal lymphopoietin (TSLP), and IL-31, along with elevated Th17/IL-23 pathways and increased IgE levels [103]. In many previous experimental studies, PM has been shown to trigger molecular pathways associated with the pathogenesis of AD (Table 2, Figure 2). In mouse models, PM induces or worsens AD-like skin lesions [87,89,90]. Additionally, PM exposure has been found to increase epidermal thickness and promote the infiltration of dermal inflammatory cells, including mast cells, which are characteristic histopathological features of AD [43,67,89]. Additionally, PM treatment upregulates the expression of various inflammatory cytokines, such as IL-1α, IL-1β, IL-4, IL-6, IL-17α, IL-25, IL-31, and TSLP, in both mouse and in vitro cell models [42,43,88]. PM also induces barrier dysfunction and increases transepidermal water loss by downregulating essential epidermal proteins, such as filaggrin and loricrin [42,43,67,86]. These proteins are crucial for skin differentiation and barrier functions, and their reduced expression compromises skin integrity. Activated AhR causes excessive epidermal innervation of transient receptor potential vanilloid 1 neurons by inducing artemin. This results in a decreased threshold for itching, leading to persistent itching and scratching, commonly observed in AD [89].

### 4.2. Psoriasis

Psoriasis is a chronic immune-mediated inflammatory skin disorder characterized by well-demarcated erythematous plaques with silvery scales that commonly affect the extensor surfaces, scalp, and nails. Epidemiological studies investigating the association between PM exposure and psoriasis have reported inconsistent results (Table 1). Several studies have found that PM2.5 and PM10 are positively associated with hospital visits for psoriasis or flare-ups [65,78,104]. In contrast, another study reported no significant effect of PM on psoriasis [79]. Further studies are required to quantify the clinical effects of PM exposure on psoriasis.

The pathogenesis of psoriasis involves a complex interplay between genetic predisposition and environmental triggers, leading to dysregulated keratinocyte proliferation and inflammatory cell infiltration, primarily mediated by T helper (Th) 17 and Th1 cells [105]. Differentiated and activated Th17 cells secrete IL-17A, stimulating keratinocytes and causing epidermal hyperproliferation in psoriasis [106]. Keratinocytes then release antimicrobial peptides, such as LL-37 and S100-alarmins [107]. Notably, S100a7 (psoriasin) and S100a8 (calgranulin A) are highly upregulated in psoriatic lesions, driving dysregulated keratinocyte differentiation, neutrophil chemotaxis, abnormal angiogenesis, and increased inflammation [108,109]. In a mouse model, PM2.5 exposure induced more severe psoriatic skin lesions and increased the expression of keratin 17 protein as well as S100a8 and S100a7a [91]. In addition, protein kinase B (Akt)/mammalian/mechanistic target of rapamycin (mTOR)/hypoxia-inducible factor (HIF)-1α/vascular endothelial growth factor (VEGF) signaling pathways were highly activated in psoriatic skin after PM2.5 exposure (Table 2, Figure 2). In the in vitro model, a specific agonist of AKT (740Y-P) reversed the decreased neovascularization induced by KRT17 knockdown through the AKT/mTOR/HIF-1α signaling pathway in vitro. This suggests that PM2.5 exposure could promote the development and progression of psoriasis through KRT17-dependent activation of the AKT/mTOR/HIF-1α signaling pathway [91]. In other studies, PM disrupted SOX2 expression and keratinocyte differentiation and upregulated pro-inflammatory cytokines and psoriatic skin disease-related genes, including IL-1β, IL-6, CXCL1, CXCL2, CXCL3, CCL20, CXCL8, and S100A7 and S100A9 [92,93]. PM2.5 treatment also led to a decrease in skin barrier markers, including keratin 10, desmocollin 1, and claudin 1, in a three-dimensional skin model [93].

### 4.3. Acne

Acne is a common inflammatory skin disorder that affects the pilosebaceous unit and is characterized by comedones, papules, pustules, nodules, and cysts. It primarily occurs on the face, chest, and back, and predominantly affects adolescents and young adults. Exposure to high levels of PM has been associated with an increased number of acne outpatient visits, particularly in urban areas with significant air pollution; however, the results were not consistent across all studies (Table 1, [80,81,82,83]). Further epidemiological studies are required to elucidate the long-term effects of PM on acne.

The primary pathogenesis of acne involves increased sebum production, follicular keratinization, inflammation, and colonization by *Cutibacterium acnes* (*C. acnes*) [110,111]. *C. acnes* triggers the release of pro-inflammatory cytokines, such as IL-1, IL-6, IL-8, and tumor necrosis factor-alpha (TNF-α), from keratinocytes, sebocytes, and immune cells. This bacterium also activates the TLR pathways in immune and skin cells, further amplifying the inflammatory response [112]. This activation leads to the recruitment of neutrophils and other immune cells to the site, contributing to inflamed acne lesions such as papules, pustules, and nodules. To date, a few experimental studies have explored the molecular effects of PM on specific acne pathogenesis (Table 2, Figure 2). PM exposure was shown to increase the diameter and thickness of *C. acnes*-induced inflammatory nodules and upregulate inflammatory biomarkers, including IL-1α, IL-1β, IL-6, IL-8, TNF-α, matrix metalloproteinase (MMP)-1, MMP-3, and MMP-12, in a mouse model [95]. It also enhanced the expression of peroxisome proliferator-activated receptor γ, stearoyl-CoA desaturase, sterol regulatory element-binding protein (SREBP) 1a, and SREBP1c in cultured sebocytes, increasing sebum production in the mouse model [95]. In addition, PM was shown to increase the phosphorylation of NF-κB in *C. acnes*- and PGN-treated keratinocytes, and increase COX2 and TLR4 levels [94]. This suggests that PM may exacerbate acne symptoms by amplifying the inflammatory response.

### 4.4. Aging Skin

Aging skin is characterized by structural and functional changes that lead to visible signs, such as wrinkles and pigmentation. Factors such as ultraviolet radiation, oxidative stress, and environmental pollutants accelerate the aging process. These alterations reflect the cumulative effects of intrinsic aging and extrinsic factors on skin integrity and appearance. Several epidemiologic studies have shown that exposure to PM2.5 and PM10 is associated with increased pigmented spots and more prominent wrinkles [14,84]. Furthermore, another study demonstrated the relationship between PM2.5 and the occurrence of senile lentigo, but not with seborrheic keratosis [85]. These studies underscore the significant effect of PM on skin aging by contributing to changes in pigmentation and wrinkle formation.

The primary pathomechanism of wrinkle formation involves the degradation of extracellular matrix (ECM) proteins in the dermis, including collagen, fibronectin, elastin, and proteoglycans, leading to decreased skin elasticity and firmness. Dermal fibroblasts are responsible for the synthesis and degradation of ECM proteins. Notably, as aging progresses, collagen degradation increases while its production decreases, exacerbating the loss of skin structure and contributing to wrinkle formation [113,114,115]. Interestingly, although fibroblasts alone are not directly affected by PM exposure, they exhibit increased expression of phospho-NFκB, MMP-1, and COX2, along with decreased expression of procollagen I when co-cultured with keratinocytes (Table 2, Figure 2) [98,99]. This phenomenon can be understood within the context of “inflammaging”, a term describing the chronic, low-grade inflammation contributing to aging. When keratinocytes are exposed to PM, they initiate inflammatory responses that include the release of cytokines and other signaling molecules. These inflammatory signals can activate nearby fibroblasts, even if the fibroblasts themselves are not directly exposed to PM. The activation of NF-κB in fibroblasts, a key regulator of inflammatory responses, leads to the upregulation of MMP-1, an enzyme responsible for collagen degradation, and COX2, an enzyme involved in inflammatory processes. The reduction in procollagen I expression further exacerbates the breakdown of the extracellular matrix, contributing to a loss of skin integrity and accelerating aging.

Another key aspect of skin aging is pigmentation changes, such as age spots and uneven skin tone, which result from the overproduction and uneven distribution of melanin. These changes are often exacerbated by sun exposure and photoaging [116]. PM has been shown to increase melanin production in human keratinocytes, in vivo mouse models, and ex vivo human skin models [96,97]. This process is associated with the upregulation of the inositol-requiring enzyme type 1 (IRE1) α signaling pathway, indicating an association with ER stress [96] (Table 2, Figure 2). Furthermore, one study showed that PM exposure increased the mRNA expression of melanogenic cytokines, such as stem cell factor and endothelin-1, in keratinocytes and enhanced melanin synthesis in a keratinocyte:melanocyte (1:1) co-culture [97]. This indicates that melanocytes alone or in combination with keratinocytes increase melanin production when exposed to PM, leading to increased skin pigmentation.

### 4.5. Summary

Numerous epidemiological studies have demonstrated a relationship between various inflammatory skin diseases, such as AD, eczema, psoriasis, acne, and aging skin, and PM exposure. At the molecular level, PM exposure exacerbates AD by inducing inflammatory responses, barrier disruption, and pruritus. In psoriasis, PM disrupts keratinocyte differentiation and upregulates pro-inflammatory cytokines. In acne, PM enhances inflammatory biomarkers and increases sebum production in inflammatory nodules. Regarding skin aging, PM degrades ECM proteins and upregulates MMP-1 and COX2 in fibroblast-keratinocyte co-cultures. Additionally, PM boosts melanin production through the IRE1α pathway and increases melanogenic cytokines, leading to increased skin pigmentation.

## 5. Discussion

The molecular impact of PM on skin diseases is an area of growing interest, with many studies highlighting the complex interactions between environmental pollutants and dermatological conditions. However, while the evidence linking PM exposure to skin diseases such as AD, psoriasis, acne, and skin aging is compelling, several limitations in the existing research need to be addressed to understand these mechanisms and their implications for public health fully.

One of the primary limitations of current studies is the variability in experimental models used to study the effects of PM. Many studies rely on in vitro models or animal studies, which, while valuable, may not fully capture the complexity of human skin responses to PM. Additionally, these models often use isolated components of PM, which may not reflect the full spectrum of environmental pollutants. Another limitation is the lack of long-term studies assessing the chronic effects of low-level PM exposure, which is more representative of real-world conditions. Most studies have focused on acute, high-dose exposures, which may not accurately depict the cumulative impact of PM on skin over time. Furthermore, there is limited research on the interaction between PM and other environmental factors, such as ultraviolet irradiation, temperature, and humidity, which could modulate the effects of PM on the skin. The role of individual genetic susceptibility in response to PM exposure remains underexplored, particularly in different skin types and conditions.

To address these gaps, future research should develop more sophisticated in vitro and in vivo models that closely mimic human skin, incorporating the full range of environmental factors that influence the effects of PM. Long-term epidemiological studies are needed to understand better the chronic effects of PM exposure on skin health, especially in populations with varying levels of exposure and genetic backgrounds. Additionally, research should aim to elucidate how PM interacts with other environmental factors, such as UV radiation, to exacerbate skin conditions.

One emerging topic for future research is exploring the skin microbiome’s role in modulating PM’s effects. It has been shown that the composition of the skin microbiome is dependent on the living environment (polluted and less polluted) [117,118,119]. Furthermore, these changes have been correlated with skin pigmentation dysfunction in individuals residing in polluted environments, highlighting the impact of pollution on skin health and the microbiome [117]. Given the interplay between the skin microbiome and PM, targeting the microbiome presents a novel therapeutic approach to mitigating the effects of PM on the skin. Probiotic and prebiotic treatments, designed to restore a healthy microbiome balance, could help strengthen the skin’s barrier function and reduce inflammation. For instance, anti-pollutant treatment strategies targeting the microbiome could involve beneficial bacteria like *Roseomonas mucosa*, which has shown promise in treating AD by modulating lipid production and TNF signaling. This approach could also have potential in psoriasis, where *R. mucosa* has demonstrated preclinical efficacy, suggesting that microbial manipulation may offer therapeutic benefits in managing skin diseases exacerbated by environmental pollutants, including PM [120]. Additionally, understanding the specific microbial changes induced by PM exposure could lead to developing personalized skincare products that cater to individual microbiome profiles. This personalized approach could optimize the skin’s resilience against environmental pollutants by supporting a healthy and balanced microbiome.

In conclusion, while significant progress has been made in understanding the molecular impact of PM on skin diseases, there is still much to be explored. Addressing the limitations of previous studies through more advanced research methods, exploring newly emerging molecular targets, and considering potential therapeutic strategies will be crucial in developing practical approaches to mitigate the harmful effects of PM on skin health.

## Figures and Tables

**Table 1:** Summary of epidemiological studies on the association between outdoor particulate matter and skin diseases.

Skin Disease	Study	Location	PM	Design	Age (Y)	Sample Size (no.)	Major Findings
Atopic dermatitis (AD)	Gu et al., 2023 [61]	UK	PM2.5 PMcoarse PM10	prospective cohort study	40–70	337,910	The medium (HR, 1.182, *p* = 0.003) and high (HR, 1.359, *p* < 0.001) air pollution mixture was significantly associated with incident AD compared with the low pollution group. PM2.5 absorbance accounted for the majority proportion of effects for air pollution mixtures.
	Keller et al., 2022 [62]	USA	PM2.5	Cohort study	≤ 18	16,089,050	PM2.5 was not associated with atopic eczema when adjusted for NO_2_.
	Park et al., 2022 [63]	South Korea	PM2.5 PM10	Population-based retrospective cohort study	0–75	209,168	PM2.5 (HR, 1.420; 95% CI, 1.392–1.448; for 1 μg/m^3^) and PM10 (HR, 1.333, 95% CI, 1.325–1.341; for 1 μg/m^3^) showed a significant positive association with AD incidence.
	Ye et al., 2022 [64]	China	PM2.5 PM10	Time-series study	All	34,633	Exposure to PM2.5 and PM10 worsened symptoms of AD, with PM10 being more detrimental than PM2.5. Children aged 0–7 years and women were the most vulnerable to particulate matter.
	Park et al., 2021 [65]	South Korea	PM2.5 PM10	Time-series study	All	23,288,000	A 10 μg/m^3^ increase in PM2.5 and PM10 led to patient visit increases of 2.71% (95% CI: 0.76–4.71; *p* < 0.01) and 2.01% (95% CI: 0.92–3.11; *p* < 0.001), respectively.
	Wang et al., 2021 [66]	China	PM2.5 PM10	Time-series study	All	45,094	An increase of 10 μg/m^3^ in PM2.5 was associated with a 1.1% (95% CI: 0.6%–1.7%) rise in dermatitis/eczema visits at lag 0–1 days.
	Bae, et al., 2020 [67]	South Korea	PM2.5 PM10	Time-series study	All	513,870	The risk of AD-related visits was associated with higher PM10 (RR, 1.009, 95% CI: 1.007–1.012).
	Tang et al., 2017 [68]	Taiwan	PM2.5 PM10	Cross-sectional study	≥ 20	1023	PM2.5 was significantly associated with AD (aOR, 1.05, 95% CI:1.02–1.08).
	Kim et al., 2017 [69]	South Korea	PM10	Panel study	0–5	177	A 10 μg/m^3^ increase in PM10 was associated with a 3.2% (95% CI: 1.5–4.9%) increase in visits on the same day.
	Liu et al., 2016 [70]	China	PM10	Cross-sectional study	4–6	3358	No significant association between PM10 and childhood eczema.
	Wang et al., 2016 [71]	China	PM2.5 PM10	Cross-sectional study	Kindergarten children	2661	No significant association.
Eczema	Sun et al., 2022 [72]	China	PM10	Cross-sectional study	Preschool age	2399	PM10 concentrations were positively associated with eczema symptoms (*p* = 0.03).
	Hüls et al., 2019 [73]	Germany	PM2.5 PM10	Cohort study	≥55	834	Baseline PM10 and PM2.5 levels were associated with incidence of nonatopic eczema (OR, 1.356, 95% I: 1.004–1.831, OR, 1.454, 95%CI: 1.064–1.987).
	Wang et al., 2019 [74]	China	PM2.5	Time-series study	≥18	1,966,991	A 10 μg/m^3^ increase in PM2.5 on the current day was associated with a 0.30% (95% CI: 0.28–0.33%) change in the number of hospital visits.
	Li et al., 2018 [75]	China	PM10	Time-series study	All	14,000,000	An increase of 10 μg/m^3^ in PM10 corresponded to an increasing RR of 1.0024 at lag 0, which represented an increase of 0.24% in eczema outpatient visits.
	Shah et al., 2016 [76]	US	PM10	Cross-sectional study	3 m–58 m	128	A comparison of subjects with and without eczema showed a difference in the natural log of PM collected from the PIPER air sampling (*p* = 0.049).
Psoriasis	Bellinato et al., 2022 [77]	Italy	PM2.5 PM10	Cross-over and cross-sectional study	All	369	Exposure to mean PM10 over 20 μg/m^3^ and mean PM2.5 over 15 μg/m^3^ in the 60 days before assessment were associated with a higher risk of PASI 5 or greater point worsening (aOR, 1.55, 95% CI: 1.21–1.99 and aOR, 1.25, 95% CI: 1.0–1.57).
	Wu et al., 2022 [78]	China	PM2.5	Time-series study	All	500,266	A same-day increase of 10 μg/m^3^ in PM2.5 concentrations was associated with a 0.29% (95% confidence interval: 0.26–0.32%) increase in daily outpatient visits for psoriasis. Female and older patients appeared to be more sensitive to the effects of PM2.5 (*p* < 0.05).
	Lee et al., 2022 [79]	Korea	PM2.5 PM10	Longitudinal study	All	Poulation of 7 major cities in Korea	PM2.5 and PM10 had no impact on hospital visits of psoriasis patients.
	Park et al., 2021 [65]	Korea	PM2.5 PM10	Time-series study	All	23,288,000	Concentrations of PM2.5 and PM10 were significantly associated with a increase in psoriasis patient visits, with increases of 1.46% (95% CI: 0.53–2.40%; *p* < 0.01) and 1.50% (95% CI: 0.99–2.02%; *p* < 0.001), respectively.
Acne	Li et al., 2022 [80]	China	PM2.5	Time-series study	All	120,842	A 10 μg/m^3^ increase in PM2.5 concentration at lag 0–7 was associated with a 1.71% (95% CI: 1.06–2.36%) rise in acne outpatient visits, with individuals aged 25 and older being more susceptible than those under 25 years.
	Li et al., 2021 [81]	China	PM10	Time-series study	All	71,625	The association between PM10 and acne visits was significant only in middle-aged and older adults (over 30 years old), with a 10 μg/m^3^ increase in PM10 at lag 0–3 corresponding to a 0.46% (95% CI: 0.08–0.84%) increase in acne visits.
	El Haddad et al., 2021 [82]	Lebanon	PM2.5 PM10	Cross-sectional study	18–55	372	No association was found between PM2.5, PM10, and acne.
	Liu et al., 2018 [83]	China	PM2.5 PM10	Time-series study	All	59,325	PM10 and PM2.5 showed significant effects on the number of outpatient visits for acne vulgaris after additionally adjusting for SO2 (PM10: RR, 1.023, *p* < 0.05; PM2.5: RR, 1.016, *p* < 0.05).
Skin Aging	Huang et al., 2022 [84]	Taiwan	PM2.5 PM10	Cross-sectional study	30–74	389	High PM10 and PM2.5 concentrations were both associated with high brown spot scores (coefficient β, 0.48, *p* = 0.00, and coefficient β, 0.86, *p* = 0.00, respectively).
	Peng et al., 2017 [85]	China	PM2.5	Cross-sectional study	40–90	400	People living in districts with low PM2.5 levels tended to have a lower number of spots on their cheeks and the backs of their hands (OR, 0.403, 95% CI: 0.239–0.679; OR, 0.263, 95% CI: 0.146–0.441). No association with seborrheic keratosis.
	Vierkotter et al., 2010 [14]	Germany	PM2.5 PM10	Cross-sectional study	70–80	400	There were 22% more spots on the forehead and 20% more spots on the cheeks per increase of one interquartile range (IQR) of PM2.5 absorbance (OR, 1.22, 95% CI: 1.03–1.45, OR, 1.20, 95% CI: 1.03–1.40).

PM = particulate matter, OR = odds ratio, aOR = adjusted odds ratio, RR = relative risk, HR = hazard ratio, CI = confidence interval.

**Table 2:** Summary of the impact of PM on specific skin conditions at a molecular level.

Skin Disease	Study	Model	PM Type	PM Application	Main Findings
Atopic dermatitis (AD)	Roh et al., 2024 [42]	AD-like triple cell model	SRM 1649b	₋ 25 μg/cm^2^, systemic	₋ ↑ IL-6, IL-1β, and IL-1α ₋ ↓ IL-10 ₋ ↓ mRNA level of filaggrin and loricrin
	Kwack et al., 2022 [43]	DNCB-induced AD mouse model (BALB/c)	PM_10_-like ERM71 CZ120	₋ 100 μg/mL, topical	₋ ↑ Serum IgE ₋ ↑ Epidermal thickness and mast cell count ₋ ↑ IL-1β, IL-4, IL-6, IL-17α, IL-25, IL-31 and TSLP ₋ ↓ Loricrin and filaggrin
	Kim et al., 2021 [86]	₋ Hairless mice (Crl: SKH1-Hrhr) ₋ HSE ₋ HEK	PM2.5 from Seoul, Korea	₋ 1~10 ng/mL and 100 ng/mL ₋ Topical in the mouse study, systemic in the cell study and 3D skin model	₋ ↓ FLG, loricrin, keratin-1, desmocollin-1, and corneodesmosin ₋ ↑ TEWL
	Bae et al., 2020 [67]	₋ OXA-induced AD mouse model (BALB/c) ₋ HaCaT cells	SRMs 1648a and 1649b	₋ 100 µg/cm^3^ airborne in the mouse study ₋ 50 and 25 µg/cm^2^, topical in the cell study	₋ ↑ Epidermal and dermal thickness ₋ ↑ Dermal inflammation ₋ ↓ mRNA level of filaggrin, involucrin, loricrin, claudin-1, and ZO-1
	Woo et al., 2020 [87]	₋ OVA-induced AD mouse model ₋ (BALB/c)	PM10 SRM 2787	₋ 2.5mg/mL, topical	₋ ↑ Skin severity scores, TEWL and epidermal thickness ₋ ↑ *S100A9*, *SPRR2D*, *SPRR2B*, *S100A8*, *SPRR2A3* gene expression
	Li et al., 2020 [88]	₋ Murine keratinocyte cell line PAM212	PM2.5	₋ 20, 50 and 100 μg/mL, systemic	₋ ↑ mRNA and protein level of TSLP
	Hidaka et al., 2017 [89]	₋ AhR-CA mice	Diesel exhaust particles	₋ 1mg/mL, topical	₋ ↑ AD-like lesions ₋ Epidermal hyper-innervation and inflammation ₋ Hypersensitivity to pruritus
	Sadakane et al., 2013 [90]	₋ PiCl-induced AD mouse model (NC/Nga)	Diesel exhaust particles	₋ Topical	₋ Worsening of AD-like skin lesions ₋ ↑ IL-4, keratinocyte chemoattractant, and neutrophils
Psoriasis	Wang et al., 2022 [91]	₋ Psoriasis mouse model (C57BL/6) ₋ KRT17 knockdown psoriasis cell model	PM2.5	₋ 183.80 μg/m^3^, airborne	₋ More severe psoriatic skin lesions ₋ ↑ KRT17 protein ₋ ↑ AKT/mTOR/HIF-1α signaling pathway ₋ ↑ S100a8 and S100a7a ₋ Specific agonist of AKT (740Y-P) reversed the decreased neovascularization induced by KRT17 knockdown through AKT/mTOR/HIF-1α signaling pathway
	Cheng et al., 2020 [92]	₋ hESC-based differentiation models	Ultrafine carbon nanopowder	₋ 1, 10 and 100 ng/mL, 1 and 10 µg/mL, systemic	₋ ↓ SOX2 expression ₋ ↓ Keratinocyte differentiation ₋ ↑ Inflammation and psoriasis-related genes (*IL-1β*, *IL-6*, *CXCL1*, *CXCL2*, *CXCL3*, *CCL20*, *CXCL8*, and *S100A7* and *S100A9*)
	Kim et al., 2017 [93]	₋ NHEK ₋ HSE	PM2.5	₋ 25 µg/mL, systemic in the cell study ₋ 50 µg/mL topical in the 3D skin model	₋ ↑ Pro-inflammatory cytokines and psoriatic skin disease-related genes in keratinocytes ₋ ↓ keratin-10, desmocollin 1, claudin, and IL-36γ in HSE ₋ ↑ S100A7 and S100A8 in HSE
Acne	Noh et al., 2022 [94]	₋ NHEK	SRM 1649b	₋ 10 μg/cm^2^, systemic	₋ ↑ mRNA and protein levels of proinflammatory cytokines, COX2, TLR4, and the phosphorylation of NF-κB
	Kwack et al., 2022 [95]	₋ Human sebocytes and ORS cell ₋ Acne mouse model (HR-1)	PM10-like (ERM-CZ120)	₋ 100 μg/mL, systemic in the cell study and topical in the animal study	₋ IL-1α, IL-1β, IL-6, IL-8, TNF-α, MMP-1 in sebocytes and ORS cells ₋ ↑ *C. acnes*-induced inflammatory nodule diameter and thickness in a mouse model ₋ ↑ IL-1α, IL-1β, IL-6, IL-8, TNF-α, MMP-1, MMP-3, and MMP-12 in inflammatory nodules in the mouse model ₋ ↑ sebum production in inflammatory nodules in the mouse model ₋ ↑ PPARγ, SCD, SREBP1a, and SREBP1c in cultured sebocytes
Skin aging	Ahn et al., 2022 [96]	₋ C57BL/6 mice ₋ NHM ₋ HSE	₋ SRM 1648a	₋ 25 μg/mL, topical in the mouse study, systemic in the cell study and 3D skin model	₋ ↑ Melanin production in melanocytes, mouse model, and human skin model ₋ ↑ IRE1α signaling pathway
	Moon et al., 2022 [97]	₋ NHM ₋ NHK ₋ HSE ₋ HDF	₋ PM10, from Seoul, Korea ₋ SRM 1648a and 1649b	₋ 100 μg/mL, systemic	₋ ↑ mRNA level of IL-1α, IL-1β, IL-8, and MMP-3 in in keratinocytes ₋ ↑ melanin synthesis in keratinocyte: melanocyte (1:1) co-culture ₋ ↑ Epidermal melanin pigmentation in ex vivo skin tissue ₋ ↑ mRNA level of SCF and ET-1 in keratinocytes ₋ ↑ mRNA level of MMP-1, MMP-2, and MMP-3 in dermal fibroblasts treated with the conditioned media obtained from keratinocytes exposed to local PM10
-	Ko et al., 2020 [98]	₋ NHM ₋ HaCaT cells ₋ HDF	PM2.5 (NIST 1650b)	₋ 50 ppm, systemic	₋ ↑ ET-1 and PGE2 in keratinocyte/melanocyte coculture ₋ ↑ NF-κB, cysteine-rich protein 61, and MMP-1, ↓ Procollagen type I in fibroblast/keratinocyte co-culture
	Kim et al., 2019 [99]	₋ HaCaT cells ₋ HDF	SRM 1648a and 1649b	₋ 50 μg/cm^2^ for 1648a and 25 μg/cm^2^ for 1649b, systemic	₋ ↑ IL-1α and IL-1β in keratinocytes ₋ ↑ MMP1 and COX2 in fibroblasts co-culture with keratinocytes

PM = particulate matter, IL = interleukin, DNCB = 1-chloro-2,4-dinitrobenzene, OXA = oxazolone, OVA = ovalbumin, HaCaT cell = human, adult, low calcium, high temperature, HEK = human epidermal primary keratinocyte, CA-AhR = constitutively active aryl hydrocarbon receptor, KRT17 = keratin 17, hESC = human embryonic stem cell, HSE = human skin equivalent, NHEK = normal human epidermal keratinocyte, ORS = outer root sheath, NHM = normal human epidermal melanocyte, NHK = normal human epidermal keratinocyte, HDF = human dermal fibroblast, MMP = matrix metalloproteinase, FLG = filaggrin, TEWL = transepidermal water loss, ZO-1 = zonula occludens-1, TSLP = thymic stromal lymphopoietin, AKT = protein kinase B, mTOR = mammalian target of rapamycin, HIF-1a = hypoxia inducible factor 1 subunit alpha, SOX-1 = SRY-Box transcription factor 1, CCL, CXCL = chemokine ligand, COX2 = cyclooxygenase, TLR = toll-like receptor, NF-κB = Nuclear factor kappa B, PPARγ = peroxisome proliferator-activated gamma receptors, SREBP = Sterol regulatory element binding proteins, IRE1α = Inositol-requiring transmembrane kinase/endoribonuclease 1α, SCF = stem cell factor, ET-1 = endothelin 1, PGE = prostaglandin E.

## Data Availability

Data sharing not applicable—no new data generated.
